# Stable Isotopes Reveal the Drivers of Post‐Wildfire Natural Regeneration of Interior Douglas‐Fir Seedlings in British Columbia

**DOI:** 10.1002/ece3.71078

**Published:** 2025-03-13

**Authors:** Julie McAulay, José Ignacio Querejeta, Gabriel Danyagri, Bianca N. I. Eskelson, Timothy J. Philpott, Sari C. Saunders, Eliot Mompeán, Ignacio Barbeito

**Affiliations:** ^1^ Department of Forest Resources Management The University of British Columbia Vancouver British Columbia Canada; ^2^ Department of Soil and Water Conservation CSIC‐Centro de Edafología Y Biología Aplicada del Segura Murcia Spain; ^3^ B.C. Ministry of Forests Williams Lake British Columbia Canada; ^4^ B.C. Ministry of Forests Coast Research Nanaimo British Columbia Canada

**Keywords:** carbon stable isotopes, early successional dynamics, foliar nutrients, nitrogen stable isotopes, oxygen stable isotopes, water stress, water use efficiency

## Abstract

Wildfires are increasing in frequency and severity due to climate change, posing challenges to forest ecosystems, including the southern interior of British Columbia, Canada. Interior Douglas‐fir ( *Pseudotsuga menziesii var. glauca* ) is a species of great cultural, ecological, and economic importance, necessitating the investigation of post‐wildfire regeneration amidst this changing wildfire regime. This study examines interior Douglas‐fir seedling regeneration across three burn severity levels (low, moderate, high) 5 years post‐wildfire at a site in interior British Columbia. Natural regeneration and seedling traits were measured in 2022 and paired with stable isotope analyses (δ^13^C, δ^15^N, δ^18^O) and foliar nutrient assessments. We employed linear mixed‐effects models to assess the impact of burn severity and light, water, and nutrient factors on seedling biomass. Results indicate higher seedling density in low severity sites but larger individual biomass in moderate and high severity sites. Light availability was the primary factor limiting individual seedling biomass, with greater δ^13^C and biomass in high severity sites, suggesting that reduced canopy cover enhances photosynthesis and water use efficiency. Despite higher solar exposure, seedlings in high severity sites did not show increased drought stress according to leaf δ^18^O and stem water contents, likely due to reduced interception and competition for soil water by overstory trees. Biomass growth was not linked to leaf nutrient status, indicating nutrient availability, particularly N, did not limit seedling biomass. While light availability is the current primary growth‐limiting factor for regenerating interior Douglas‐fir seedlings in this study, increased frequency and intensity of heat waves and droughts associated with climate change may increase water stress, emphasizing the need for long‐term monitoring and adaptive management to support the regeneration of interior Douglas‐fir.

## Graphical abstract

This study investigates the post‐wildfire regeneration of interior Douglas‐fir seedlings in British Columbia across different burn severity levels, focusing on the effects of light, water, and nutrient availability. Results indicate that light is the primary factor limiting seedling growth, and water availability and nutrients, particularly nitrogen, did not appear to limit biomass growth. However, future climate change‐related heat waves and droughts may increase water stress, necessitating ongoing monitoring and adaptive management strategies.

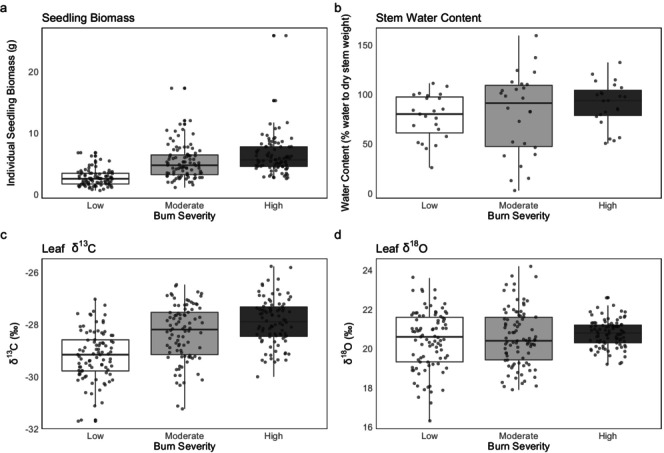

## 1 Introduction

Fire plays a key role in shaping ecological communities worldwide (Pausas and Keeley 2009; Pausas et al. 2017; He et al. 2019), but climate change is altering wildfire regimes. In Canada, the frequency of large crown fires has doubled over five decades, impacting millions of hectares, with the trend especially notable in Western Canada (Hanes et al. 2019). British Columbia (bc) historically experienced mixed‐severity fires that created diverse landscapes (Copes‐Gerbitz et al. 2022). However, fire suppression post‐European colonization led to fuel buildup in bc's dry interior forests (Copes‐Gerbitz et al. 2022), while climate change and mountain pine beetle outbreaks have further increased fire risk (Kirchmeier‐Young et al. 2019; Perrakis et al. 2014; Haughian et al. 2012).

Interior Douglas‐fir ( *Pseudotsuga menziesii var. glauca* ), a dominant species in interior bc, contributes to habitat biodiversity, carbon storage, and timber production (Government of British Columbia Ministry of Forests n.d.). Historically, interior Douglas‐fir forests in bc experienced frequent ground fires (return intervals < 50 years) and occasional stand‐replacing fires (~250 years), creating multi‐aged stands (Brookes et al. 2021). Interior Douglas‐fir typically regenerates well after ground fires due to increased light availability and reduced competition for soil resources (Vyse et al. 2006; Barker et al. 2014), but successful seedling establishment depends on many factors like light, plant community structure, seed source proximity, soil properties, mycorrhizal access, moisture, and suitable substrate (Young et al. 2019; Kemp et al. 2016; McDowell et al. 2011). While some studies across western North America indicate enhanced regeneration for combined interior and coastal Douglas‐fir (Povak et al. 2020) and seedling biomass accumulation for interior Douglas‐fir (Barker et al. 2014) on moderate to high burn severity sites, microclimatic variations can substantially affect seedling performance (Ortiz et al. 2019).

Most previous studies of post‐fire Douglas‐fir regeneration largely focus on seedling density (seedlings per unit area), occurrence (% plots with species present) (Povak et al. 2020) or biomass (average individual seedling in grams) (Barker et al. 2014), providing an overview of early recovery. However, understanding the physiological mechanisms driving individual seedling responses to mixed‐severity wildfires is crucial for climate adaptation strategies. Stable isotope analyses help pinpoint key environmental factors (light, water, nutrients) contributing to variations in seedling biomass across burn severity levels.

Stable isotope analyses measure the ratios of natural abundance isotopes in environmental samples and are a powerful tool in ecological research (Dawson et al. 2002). In order to examine seedling ecophysiology—the impact of environmental factors on seedling function, this study used stable isotope analyses of carbon, oxygen, and nitrogen isotopes (δ^13^C, δ^18^O, and δ^15^N, respectively) in seedling leaves and stem water. These analyses can provide insights into key physiological processes of seedlings (Marañón‐Jiménez et al. 2013; Querejeta et al. 2008; Leverkus et al. 2015; Herrero et al. 2013). Leaf δ^13^C provides information on time‐integrated intrinsic water‐use efficiency (WUEi, defined as the ratio photosynthetic rate (*A*)/stomatal conductance(*g*_*s*_)) (Farquhar et al. 1989; Dawson et al. 2002). Leaf δ^18^O, influenced by source water isotopic composition, provides information on time‐integrated stomatal conductance and cumulative transpiration (Farquhar et al. 2007; Barbour 2007). Simultaneous leaf δ^18^O and δ^13^C data help differentiate between biochemical and stomatal limitations to photosynthesis (Scheidegger et al. 2000; Siegwolf et al. 2023). Leaf δ^15^N can provide information on nitrogen cycling and sources, as well as mycorrhizal relationships (Craine et al. 2009; Hobbie and Högberg 2012; Ruiz‐Navarro et al. 2016), while also indicating ^15^N enrichment after wildfires (Huber et al. 2013; LeDuc et al. 2013). These techniques help investigate how burn severity affects environmental factors like light, water, and nutrients within microsites—small, localized areas with varying conditions, often created by wildfires—and pinpoint the environmental factors most influencing seedling growth (Marcolin et al. 2019).

We postulate that: (H1) Increased sunlight at ground level in high burn severity sites enhances photosynthesis, leading to higher leaf δ^13^C and seedling biomass; (H2) regenerating seedlings face increasing drought stress and water limitations with higher burn severity due to greater solar exposure, reduced overstory canopy shading, and drier microsites; (H3) enhanced soil nutrient availability in high‐severity sites, resulting from wildfire‐induced mineralization and the release of nutrients from forest biomass, further boosts seedling nutrient status and biomass. Understanding how burn severity affects seedling physiology will provide insights into regeneration dynamics post‐wildfire and inform effective post‐fire management strategies.

## 2 Material and Methods

### 2.1 Study Area

**FIGURE 1:**
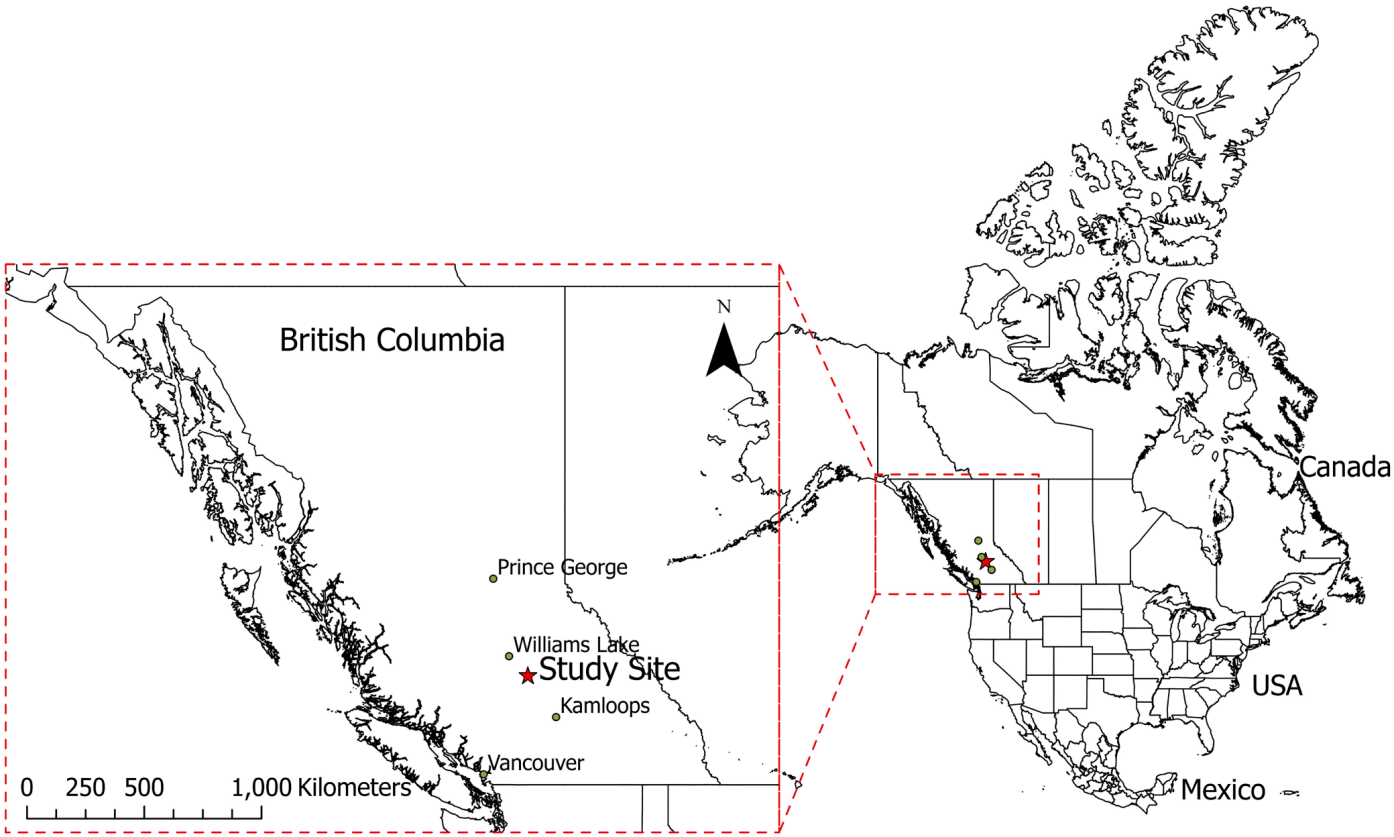
Map of study site in bc, Canada.

The study site (51.65°N, 121.47°W) is located near the town of Williams Lake in central bc (Figure 1). Elevation ranges from 1067 to 1180 m above sea level, with a mean annual temperature of 4.1°C and mean annual precipitation of 433 mm (1981–2010, Wang et al. 2016). Soils are Orthic Gray Luvisols with moderately alkaline morainal parent materials (Valentine and Schori 1980; Soil Classification Working Group 1998). This area is dominated by interior Douglas‐fir ( *Pseudotsuga menziesii var. glauca* ) with some Lodgepole pine (*Pinus contorta Dougl. var. latifolia Engelm*) present (Steen and Coupé 1997).

The Gustafson fire burned 5700 ha, including the study site, in July 2017, leaving stands with varying burn severity levels (Figure S1) (Cariboo Regional District 2018). The area was classified into low, moderate, and high burn severity classes using the differenced Normalized Burn Ratio (dNBR) (Appendix A), derived from near‐infrared (NIR) and short‐wave infrared (SWIR) bands from pre‐ and post‐fire LANDSAT‐8 (30 m resolution) and SENTINEL‐2 (20 m resolution) imagery.

**FIGURE 2:**
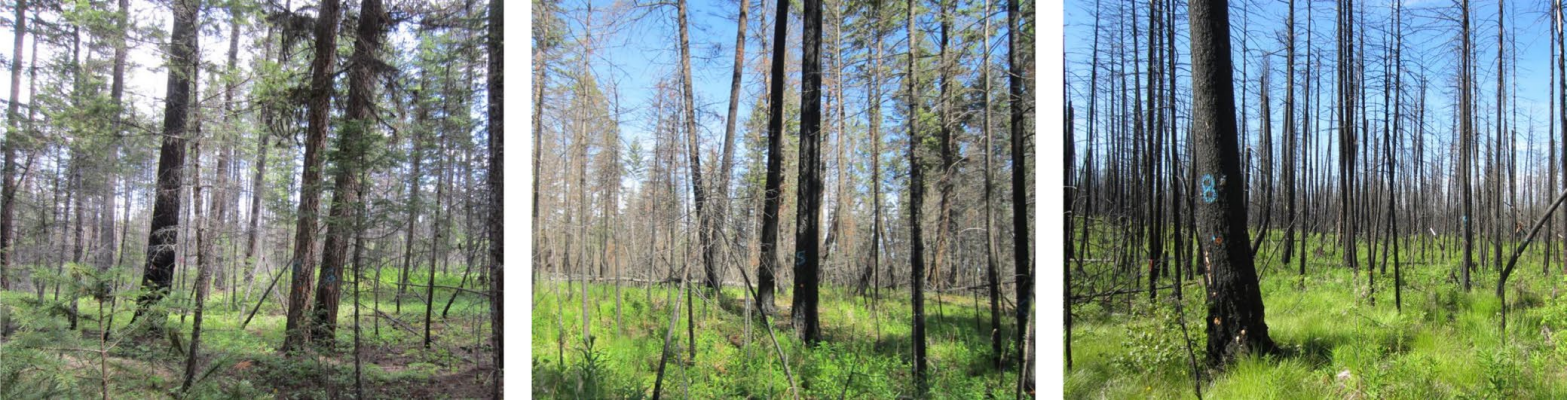
Photos of low, moderate, and high severity sites taken in 2022 (from left to right) after the 2017 wildfire.

The study was conducted in three separate blocks, each containing low, moderate, and high burn severity classes (Figure 2), resulting in three replicates per severity (*n* = 9 treatment units). Within each burn severity class in each block, three 11.28 m radius permanent sample plots (PSPs) were established, spaced 50 m apart. These three PSPs measured within each severity class are considered to be subsamples in our study. Their locations were randomly selected based on the middle slope position from the Digital Elevation Model of the study area. High severity PSPs were set 5 m from unburned stands to ensure seed source access. Four 1.26 m radius natural regeneration subplots were established at the PSP circumference in cardinal directions, totaling 108 subplots across 27 PSPs.

### 2.2 Field Data Collection

Natural regeneration densities of interior Douglas‐fir were recorded in each subplot and used to estimate seedling counts per hectare in 2022, 5 years post‐wildfire. Canopy cover percentage was estimated using standard lens photos taken at the center of each PSP in 2019 and analyzed with ImageJ's particle analysis function (Schneider et al. 2012).

In late August 2022, 10 naturally regenerated seedlings (3–4 years old) were randomly selected directly outside each PSP to avoid interference in long‐term regeneration monitoring. From each of the 270 seedlings, current year needles were collected, and seedling height (cm) and root collar diameter (mm) were recorded (Table 1). For three seedlings per PSP (m = 81 subsamples), total biomass (i.e., stems, needles, roots of each individual seedling; hereafter referred to as biomass) was harvested, dried, and measured for three seedlings per PSP (m = 81). For the remaining 189 seedlings, biomass was estimated using height and root collar diameter (*R* ^2^ = 0.759) (Appendix B). Twig samples were also collected and separated for this subset of 81 individual seedlings for later isotopic analysis of stem water. Mineral soil samples were collected at 0–15 cm and 15–30 cm depth intervals using a hand auger (AMS Inc., American Falls, ID, USA) in the center of each PSP (m = 54 subsamples) and placed in airtight glass vials for soil water isotopic analysis.

**TABLE 1:** All variables, sample location (within a permanent sample plot [PSP] subplot, outside of the PSP, or at the PSP center), sample size (*n*), and related growth limiting factors.

Variable	Unit	*n*	Related limiting factor
Seedling density	Seedlings ha^−1^	108	Light, water, nutrients
Seedling height	cm	270	Light, water, nutrients
Seedling diameter	mm	270	Light, water, nutrients
Seedling biomass	g	81	All
Canopy cover	%	27	Light
Leaf d13C	‰	270	Light, water
Leaf d18O	‰	270	Light, water
Stem water %	%	81	Water
Stem water d18O	‰	44	Water
Soil water d18O	‰	23	Water
Leaf d15N	‰	270	Nutrients
Leaf %C	%	270	Nutrients
Leaf %N	%	270	Nutrients
Leaf Al	mg kg^−1^	270	Nutrients
Leaf B	mg kg^−1^	270	Nutrients
Leaf Ca	g 100 g^−1^	270	Nutrients
Leaf Co	mg kg^−1^	270	Nutrients
Leaf Cu	mg kg^−1^	270	Nutrients
Leaf Fe	mg kg^−1^	270	Nutrients
Leaf K	g 100 g^−1^	270	Nutrients
Leaf Mg	g 100 g^−1^	270	Nutrients
Leaf Mn	mg kg^−1^	270	Nutrients
Leaf Na	g 100 g^−1^	270	Nutrients
Leaf Ni	mg kg^−1^	270	Nutrients
Leaf P	g 100 g^−1^	270	Nutrients
Leaf Si	mg kg^−1^	270	Nutrients
Leaf S	g 100 g^−1^	270	Nutrients
Leaf Zn	mg kg^−1^	270	Nutrients
Leaf NP ratio	Unitless	270	Nutrients
Leaf KP ratio	Unitless	270	Nutrients

*Note:* All variables were used as dependent variables with (Equation 1) to determine differences by burn severity. All variables with a related environmental factor (light, water, nutrients) were used as independent variables to investigate effects on biomass with (Equation 2). Seedling density data was collected from PSP subplots, canopy cover was collected at PSP centers, and all other variables were collected outside the PSP. All variables were sampled in 2022, except canopy cover, which was measured in 2019.

### 2.3 Leaf Isotope Composition and Nutrient Concentrations

Stable isotope analyses were performed to investigate seedling ecophysiology, with relevant variables detailed in Table 1. Foliar samples from 270 individual seedlings were oven‐dried at 60°C for 48 h, ground in a ball mill, and encapsulated (tin capsules for δ^13^C and δ^15^N, silver capsules for δ^18^O), resulting in a single sample per seedling. Carbon and nitrogen stable isotopes and elemental analyses (%C, %N) were processed at the Stable Isotope Facility, University of British Columbia, Canada, using a Thermo Scientific Delta V Plus Isotope Ratio Mass Spectrometer (IRMS) and Isoprime 100 EA. Oxygen isotopes were analyzed at the University of New Mexico Center for Stable Isotopes, USA, with a Thermo Scientific Delta V mass spectrometer. Additionally, foliar nutrient analyses (B, Ca, Cu, Fe, K, Mg, Mn, Na, Ni, P, S, Si, Zn) were conducted at CEBAS‐CSIC, Spain, using inductively coupled plasma optical emission spectrometry (ICP‐OES, Thermo Elemental Iris Intrepid II XDL) after microwave‐assisted digestion with HNO_3_:H_2_O_2_ (4:1, volume: volume).

### 2.4 Seedling and Soil Water Content

Using the subsample of 81 harvested seedlings, we collected 5–7 cm‐long basal woody twigs (main stems, 2–4 mm diameter) that were placed in air‐tight glass vials after scraping off the bark. These vials, along with 54 soil samples, were capped, wrapped with parafilm, and stored refrigerated.

Xylem and soil water were extracted using cryogenic vacuum distillation at 100°C and 10 mTorr vacuum pressure for 2 h at CEBAS‐CSIC in Spain (Ehleringer and Osmond 1989; Querejeta et al. 2021). Twig and soil water contents were measured gravimetrically by weighing samples before and after water extraction. The extracted water samples were analyzed for oxygen stable isotope ratios at the University of Lisbon, Portugal, using an Isoprime IRMS. Due to small water volumes, only 44 twig samples and 23 soil samples yielded usable results, while the remainder were discarded.

Stable isotopes are presented relative to a standard, following the formula:

$$\delta =\left(\frac{R_{\text{sample}}}{R_{\text{standard}}}-1\right)\times 1000‰$$

where R is the isotopic ratio for carbon (^13^C/^12^C), nitrogen (^15^N/^14^N), or oxygen (^18^O/^16^O). The $R_{\text{standard}}$ values used in this case were the values of Vienna Pee Dee Belemnite (VPDB) standard for carbon, the atmospheric nitrogen (AIR) standard for nitrogen, and the Vienna Standard Mean Ocean Water (VSMOW) standard for oxygen (Ehleringer and Osmond 2000).

### 2.5 Statistical Analyses

#### 2.5.1 Differences in Environmental Variables by Burn Severity

All statistical analyses were conducted using R version 4.3.2, and an *α* = 0.05 was used as the significance level for all tests (R Core Team 2023). All linear mixed effects models (LMEM) were fitted using the “lme” function in the “nlme” package in R (Pinheiro et al. 2023) and maximum likelihood estimation. We accounted for the blocking with a random block effect and treated the three plots measured within each experimental unit as subsamples, which resulted in a subsampling error and a random block effect. Although the block effect was negligible in most models (Table S1), it was retained to match the study design. All post hoc pairwise comparisons were performed with estimated marginal means using the Tukey method from the “emmeans” function in the ‘emmeans’ package (Lenth 2023).

To evaluate differences in all variables in Table 1 across different severities, the LMEM follows this form:

(1) $${\text{Variable}}_{\mathrm{ij}\left(k\right)}=\mu +R\left({\text{Block}}_{i}\right)+{Τ}_{S{\text{everity}}_{j}}+\varepsilon_{\mathrm{ij}}+\omega_{\mathrm{ij}\left(k\right)}$$

where ${\text{Variable}}_{\mathrm{ij}\left(k\right)}$ is the tested variable in block i, severity j and subsample (plot) k within each block i, μ is the overall mean of the response variable, $R\left({\text{Block}}_{i}\right)$ is the random effect factor for block i ${Τ}_{{\text{Severity}}_{j}}$ is the fixed effect factor for burn severity j, $\varepsilon_{\mathrm{ij}}$ is the experimental error term, and $\omega_{\mathrm{ij}\left(k\right)}$ is the subsampling error.

This equation was used to compare all variables (Table 1): seedling density, seedling height, seedling diameter, seedling biomass, overstory tree canopy cover, stem water content, stem water δ^18^O, soil water content, soil water δ^18^O, leaf δ^13^C and δ^18^O, and foliar nutrient variables (B, C, Ca, Cu, Fe, K, Mg, Mn, N, Na, Ni, P, S, Si, Zn, NP ratio, KP ratio) across burn severity levels. For seedling density data, we used a Generalized Linear Mixed Model (GLMM) with a Negative Binomial distribution to account for skew and overdispersion, fitted using ‘glmer.nb’ from the “lme4” package in R (Bates et al. 2015) (residual deviance = 552.8, null deviance = 571.4). Seedling biomass, diameter, height, and leaf Mn, Ni, Si, and Zn were log‐transformed, and leaf Na was square root transformed to meet normality assumptions.

### 2.6 Effects of Light, Water, and Nutrients on Seedling Biomass

To determine the light, water, and nutrient variables most important in affecting seedling biomass (all variables with associated environmental factors in Table 1), the LMEM followed this form:

(2) $$\ln\left({\text{Biomass}}_{\mathrm{ij}\left(k\right)}\right)=\mu +R\left({\text{Block}}_{i}\right)+{Τ}_{{\text{Severity}}_{j}}+{\mathrm{\beta X}}_{\mathrm{ij}}+\varepsilon_{\mathrm{ij}}+\omega_{\mathrm{ij}\left(k\right)}$$

Where $B{\text{iomass}}_{\mathrm{ij}\left(k\right)}$ is biomass in grams for block i, severity j and subsample (plot) k within each block i, μ is the overall mean of the logarithm of the response variable, $X_{\mathrm{ij}}$ is each tested variable with slope parameter β, $R\left({\text{Block}}_{i}\right)$ is the random effect factor for block i, ${Τ}_{{\text{Severity}}_{j}}$ is the fixed effect factor for burn severity j, $\varepsilon_{\mathrm{ij}}$ is the experimental error term, and $\omega_{\mathrm{ij}\left(k\right)}$ is the subsampling error. ${\text{Biomass}}_{\mathrm{ij}\left(k\right)}$ was log‐transformed to meet the linear model assumption of normality. Post hoc pairwise comparisons were performed with estimated marginal means using the Tukey method in the “emmeans” function from the “emmeans” package (Lenth 2023).

For each variable tested, the LMEM was used to evaluate whether variables related to light, water, or nutrients influenced seedling biomass (Table S2). A similar model excluding burn severity was applied to evaluate each variable's influence independently (Appendix C). Pearson correlations were used to examine the relationship between stem water content and biomass, as well as between leaf δ^13^C and δ^18^O across severity levels.

## 3 Results

### 3.1 Differences in Seedling Regeneration Across Burn Severities

**FIGURE 3:**
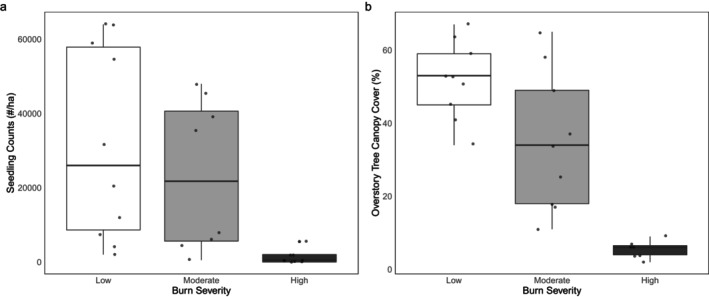
Douglas‐fir seedling counts/ha (a) taken in the subplots of each PSP and overstory tree canopy cover % at each PSP center.

**FIGURE 4:**
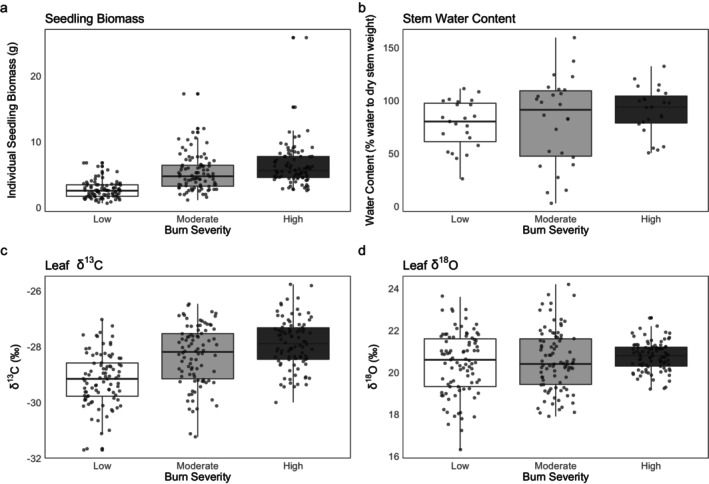
Individual seedling biomass (a), leaf δ^13^C (b), seedling stem water content (c), and leaf δ^18^O (d) of Douglas‐fir seedlings in sites of different burn severity levels.

Seedling density was lower in high burn severity sites compared to moderate (*p* = 0.0001) sites and low (*p* < 0.0001) burn severity sites (Figure 3a; Table S1). There was no significant difference (*p* = 0.884) in seedling density between low and moderate severity sites. Seedlings in high severity sites had, on average, 1.95 times greater biomass than those in low severity sites (*p* = 0.0004), while seedlings in moderate severity sites had 1.51 times higher biomass relative to low severity sites (*p* = 0.0252) (Figure 4a; Table S1). There was no significant difference in individual seedling biomass between moderate and high severity sites (*p* = 0.202). Thus, low severity sites had more seedlings per hectare, while moderate and high severity sites had larger seedling biomass.

### 3.2 Effects of Light Availability on Seedling Biomass

Light availability, indicated by overstory tree canopy cover, was significantly higher in high burn severity sites compared to low and moderate sites (*p* < 0.0001 for both) (Figure 3b; Table S1). Canopy cover also differed significantly between low and moderate severity sites (*p* = 0.0116) (Figure 3b). In the individual LMEM (Table S2), higher leaf δ^13^C values were associated with higher individual seedling biomass values, both with and without burn severity included in the model (*p* < 0.0001 for both). Canopy cover did not significantly affect seedling biomass with severity included in the model (*p* = 0.338). However, in the univariate model without severity, increased canopy cover was associated with significant decreases in seedling biomass (*p* < 0.0001) (Table S2). Leaf δ^13^C was the best predictor of individual seedling biomass of all variables tested in the model, and δ^13^C alone explained 18% of the variance in biomass across burn severities.

### 3.3 Effects of Water Availability on Seedling Biomass

There were no significant differences (*p* > 0.3) in stem water content (% water/dry tissue) (Figure 4b) or soil water content (% water/dry material) across severities, but stem water content did have a moderate, positive relationship with seedling biomass (Pearson's *r* = 0.328; *p* = 0.0049), and increases in stem water content were associated with significant increases in seedling biomass (*p* = 0.0130) (Table S2). Stem water δ^18^O values (m = 44 subsamples) did not significantly differ across burn severities (*p* = 0.114), nor did soil water δ^18^O values (m = 23) (*p* = 0.976) (Figure S2; Table S1). Average stem water δ^18^O (−7.70‰) was more similar to average 0–15 cm topsoil water δ^18^O (−8.33‰) than average 15–30 cm soil water δ^18^O (−13.6‰), and average soil water content in the 0–15 cm topsoil layer was 25%.

**FIGURE 5:**
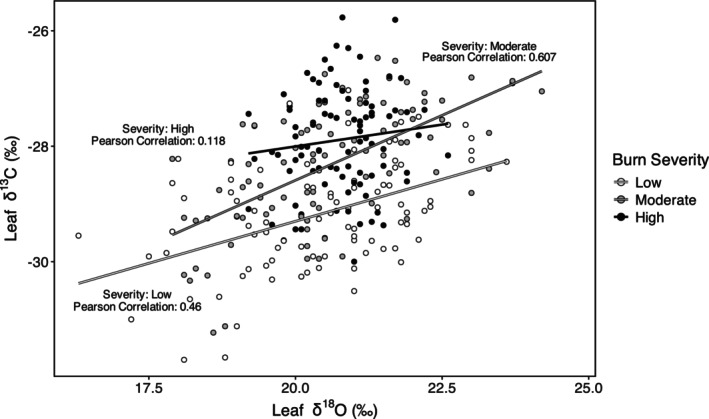
Scatterplot of leaf δ^13^C and δ^18^O values (m = 270 subsamples) for individual Douglas‐fir seedlings across burn severities in south central bc.

Leaf δ^13^C values significantly differed among burn severity treatments (*p* < 0.0001), while δ^18^O values did not (*p* = 0.675) (Figure 4c,d; Table S1). Leaf δ^13^C was, on average, 1.3‰ higher in high severity sites compared to low severity sites (*p* < 0.0001) and 0.8‰ higher in moderate severity sites compared to low severity sites (*p* = 0.0283) (Figure 4c). There was no significant difference in leaf δ^13^C between high and moderate severity sites (*p* = 0.336) (Figure 4c). There was a positive correlation between leaf δ^13^C and δ^18^O (Pearson's *r* = 0.445; *p* < 0.0001), varying in strength across burn severity levels (Figure 5).

### 3.4 Effects of Nutrients on Seedling Growth

**FIGURE 6:**
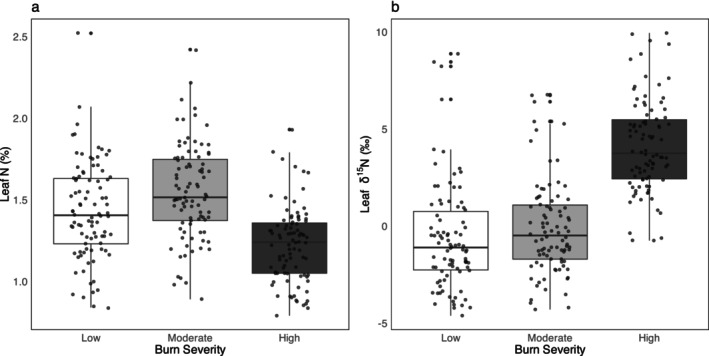
Boxplots of leaf %N (a) and δ^15^N (b) by burn severity for individual Douglas‐fir seedlings across burn severities.

In high severity sites, leaf nitrogen concentration was, on average, 0.3% lower compared to moderate sites (*p* < 0.0001) and 0.2% lower compared to low severity (*p* = 0.0039) sites, with no significant difference between moderate and low (*p* = 0.1040) (Figure 6a; Table S1).

For foliar nutrients, Ca, Fe, Mg, Mn, Si, S, Zn, and NP ratio all differed significantly across severities (*p* < 0.048), but other nutrients (B, C, Cu, K, Na, Ni, and P) did not (*p* > 0.168) (Figure S3; Table S1), and no nutrients significantly affected biomass (Table S2). Leaf NP ratios were low across all burn severity sites (average 7.3 ± 2.3 across individuals, m = 270). Leaf δ^15^N was, on average, 4.8‰ higher in high severity sites compared to low severity sites (*p* < 0.0001) and 4.2‰ higher compared to moderate severity sites (*p* < 0.0001). Leaf δ^15^N did not differ between low and moderate severity sites (*p* = 0.643) (Figure 6b; Table S1).

## 4 Discussion

### 4.1 Light Was the Key Growth Limiting Factor for Regenerating Douglas‐Fir Seedlings

Reduced overstory canopy cover in high burn severity sites increases understory sunlight availability, enhancing photosynthesis in seedlings, as indicated by higher leaf δ^13^C values and biomass growth (Scheidegger et al. 2000; Siegwolf et al. 2023). The lack of significant differences in leaf and stem δ^18^O values across burn severity levels suggests similar time‐integrated stomatal conductance and cumulative transpiration, despite higher δ^13^C values with decreasing canopy cover (Barbour 2007; Siegwolf et al. 2023). This indicates that differences in leaf δ^13^C among burn severity sites were primarily driven by enhanced photosynthesis resulting from decreased canopy cover and increased sunlight availability, as outlined in the dual‐isotope model by Scheidegger et al. (2000) and updated by Siegwolf et al. (2023). Thus, light availability emerges as the key limiting factor in this study for the growth of interior Douglas‐fir seedling individuals, confirming H1 and explaining why seedlings in high severity sites with the lowest overstory canopy cover exhibited the highest intrinsic water use efficiency (inferred from δ^13^C) and biomass growth. This result aligns with other studies showing that increased light levels enhance the growth of regenerating interior Douglas‐fir individuals after disturbance (Barker et al. 2014; Lochhead and Comeau 2012; Vyse et al. 2006). However, higher irradiance at ground level also has the potential to reduce soil moisture, which can be detrimental to seedling growth (Zustovic 2015; Vyse et al. 2006), especially as climate change alters environmental conditions (Griesbauer and Green 2010).

### 4.2 No Evidence of Increased Water Stress for Regenerating Seedlings on High Severity Sites

Stem water δ^18^O values were not significantly different across the burn severities, and they were closer to δ^18^O values of soil water in the 0–15 cm topsoil layer rather than those in the 15–30 cm layer, indicating that all seedlings were likely using water of comparable isotopic composition from this topsoil layer (Dawson et al. 2002; Barbour 2007; Ding et al. 2021). Given that topsoil (0–15 cm) water content was fairly high (25%) even at the end of summer and that stem water content was not significantly different across burn severities, we conclude that soil water availability is probably not a severe growth limiting factor for Douglas‐fir seedlings at the study site during the relatively dry late summer period at this developmental stage (3–4 years after germination). Thus, despite increased exposure to solar radiation, we found no evidence of heightened drought stress or more severe stomatal constraints on photosynthesis in high burn severity sites, contrary to H2. Therefore, higher leaf δ^13^C, intrinsic water‐use efficiency, and biomass in seedlings growing at high severity sites must be primarily driven by higher photosynthesis, rather than by reduced stomatal conductance (Scheidegger et al. 2000; Siegwolf et al. 2023). Reduced underground competition for soil water from other seedlings and overstory tree roots (Devine and Harrington 2008; Devine and Harrington 2009) and reduced rainfall interception by overstory tree canopy (Teste and Simard 2008) may offset negative impacts of greater solar radiation and hotter, drier conditions at high severity sites. However, the positive correlation between leaf δ^13^C and δ^18^O across burn severities indicates high δ^13^C values were linked not only to enhanced photosynthesis but also to tighter stomatal regulation of transpiration, suggesting mild but non‐negligible water stress across severities. Although this level of stomatal closure did not significantly impact seedling biomass in this study, this correlation suggests that soil water availability could become more limiting for interior Douglas‐fir seedling regeneration in burned areas under drier climatic conditions (Zustovic 2015; Vyse et al. 2006), or for planted seedlings due to smaller root: shoot ratios (Halter and Chanway 1993).

### 4.3 Seedling Biomass Growth Was Unrelated to Leaf Nutrient Status

Low leaf N/P ratios across all burn severities suggest nitrogen is more limiting than phosphorus for Douglas‐fir seedling growth (Güsewell 2004), which is common in temperate and boreal forests (LeBauer and Treseder 2008; Du et al. 2020), including this region of interior bc (Coleman et al. 2014). However, the lack of a relationship between biomass growth and foliar nitrogen concentration indicates that nitrogen was not a severe limiting factor during this early developmental stage. The lower foliar nitrogen concentrations at high severity sites may reflect nutrient dilution due to greater leaf expansion in larger seedlings, as nitrogen concentrations tend to decline with leaf growth (Mediavilla and Escudero 2003; Manghabati et al. 2019). Typically, wildfires cause an initial pulse of soil nitrogen loss through volatilization, followed by short‐term spikes in inorganic forms like ammonium and nitrate due to enhanced mineralization (Certini 2005; Bormann et al. 2008; Homann et al. 2011). Higher leaf δ^15^N values at high severity sites do suggest faster nitrogen cycling and greater availability of inorganic nitrogen in this study (Craine et al. 2009; Ruiz‐Navarro et al. 2016), but wildfires generally deplete long‐term nitrogen pools (Bowd et al. 2019), so continued monitoring of foliar nitrogen is important. Although some foliar nutrient concentrations varied by burn severity (Table S1), they did not significantly affect individual seedling biomass. Although wildfires release nutrients such as K, P, and Ca from forest biomass (Gómez‐Rey and González‐Prieto 2014), potentially enhancing seedling growth, this study found no link between foliar nutrients and biomass. Light, and possibly water, appear to be more critical limiting factors. Further research is needed to clarify how nutrient release from wildfires impacts seedling regeneration.

### 4.4 Management Implications

While individual seedlings in high burn severity sites generally had larger biomass than those in low severity sites, climate change poses substantial risks to future post‐wildfire regeneration. Increasing wildfire frequency with shorter return intervals between wildfires could lead to more regeneration failures, as Stevens‐Rumann and Morgan (2019) found in numerous studies across the United States. Additionally, more intense and prolonged heat waves and droughts may worsen seedling water stress during early growth, hindering post‐fire recovery (Blanco‐Rodríguez et al. 2023; Hankin et al. 2019). Increasing spatial extent of high severity wildfires may result in further challenges from limited seed sources due to fewer surviving trees (Stevens‐Rumann and Morgan 2019; Simard et al. 2021). In lower severity sites where light is a key limiting factor for individual seedling growth, managers might consider practices like salvage logging to create moderate sized canopy gaps (Zustovic 2015).

Although nitrogen was not limiting to individual seedling growth in this study, repeated fires could alter nitrogen cycling and increase gaseous nitrogen losses, potentially affecting regeneration (Pellegrini et al. 2020). Climate change‐driven increases in wildfire frequency and severity may further disrupt nitrogen dynamics, necessitating further research (Smithwick et al. 2005). More frequent and severe fires could also impact mycorrhizal communities, which are essential for Douglas‐fir establishment and growth (Philpott et al. 2025; Simard 2009). Hotter and drier climatic conditions could lead to regeneration failures and shifts from conifer forests to broadleaf or shrubland vegetation (Stevens‐Rumann et al. 2022), making it crucial to monitor post‐wildfire conifer regeneration as climate change progresses.

## Author Contributions

**Julie McAulay:** conceptualization (equal), data curation (equal), formal analysis (lead), writing – original draft (lead). **José Ignacio Querejeta:** conceptualization (equal), data curation (equal), formal analysis (supporting), funding acquisition (equal), methodology (equal), resources (equal), writing – review and editing (equal). **Gabriel Danyagri:** conceptualization (equal), data curation (equal), writing – review and editing (equal). **Bianca N. I. Eskelson:** formal analysis (supporting), methodology (supporting), validation (equal), writing – review and editing (equal). **Timothy J. Philpott:** formal analysis (supporting), writing – review and editing (equal). **Sari C. Saunders:** writing – review and editing (equal). **Eliot Mompeán:** data curation (supporting), writing – review and editing (supporting). **Ignacio Barbeito:** conceptualization (equal), data curation (supporting), project administration (lead), supervision (lead), writing – review and editing (equal).

## Conflicts of Interest

The authors declare no conflicts of interest.

## Supporting information


Figure S1‐S3.

Table S1‐S2.

Appendix S1.

Data S1.

## Data Availability

The data supporting the findings of this study are available as Supporting Information.
