# Supplementary material for: Stable Isotopes Reveal the Drivers of Post‐Wildfire Natural Regeneration of Interior Douglas‐Fir Seedlings in British Columbia
Source: Ecol Evol. 2025 Mar 13;15(3):e71078. doi: 10.1002/ece3.71078 (PMC11906369; doi:10.1002/ece3.71078)
Supplement: Supplementary file 1 — Figure S1‐S3. [file ECE3-15-e71078-s002.docx]

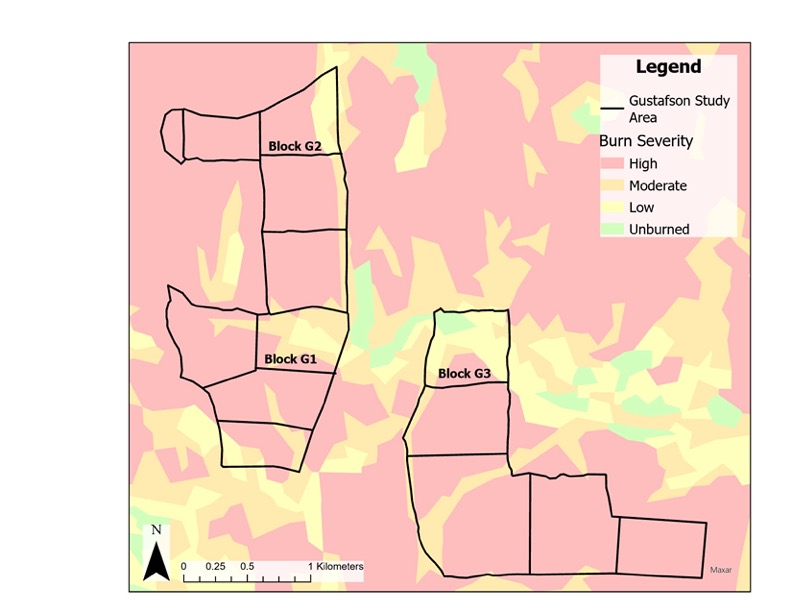


***Figure S1.*** Distribution of different burn severity levels within the three sampled blocks of the

Gustafson study area.

***Figure S2.*** Boxplots of stem water and soil water δ^18^O values by severity.

***Figure S3.*** Nutrients by burn severity.

*Note. Dashed lines indicate points of expected nutrient deficiency (Coleman et al., 2014) for the nutrients for which this information could be found.*
