# Supplementary material for: Stable Isotopes Reveal the Drivers of Post‐Wildfire Natural Regeneration of Interior Douglas‐Fir Seedlings in British Columbia
Source: Ecol Evol. 2025 Mar 13;15(3):e71078. doi: 10.1002/ece3.71078 (PMC11906369; doi:10.1002/ece3.71078)
Supplement: Supplementary file 2 — Table S1‐S2. [file ECE3-15-e71078-s003.docx]

**Table S1.** Linear mixed effect model results to compare variables in different burn severity levels

*Note. m is the number of subsamples taken for each variable.* $\hat{\sigma}_{\left( {Block}_{i} \right)}$ *is the random block effect, and* $\hat{\sigma}_{\omega_{ij(k)}}$ *is the subsampling error. G1, G2, and G3 are the individual block effects for those respective three blocks in this study. Most block effects were small, but for some variables (Leaf δ^13^C, Stem water δ^18^O),* $\hat{\sigma}_{\omega_{ij(k)}}$ *was larger than* $\hat{\sigma}_{\left( {Block}_{i} \right)}$*, indicating more variability within the block than across the blocks. Most block effects are similar, but for Height, Diameter, and Biomass, the block effect for G3 is different than those for G1 and G2, likely because this block had more unburned and low severity burn areas than the other two (Figure S1), indicating environmental differences in this block.* $x^{2}$ *is the chi squared value. Contrast values represent the differences between severity levels, calculated as simple subtractions (e.g., Low - Mod, Low - High, Mod - High). Contrast values for Na were back-transformed from a square root transformation, but remain difference values. For the values in italics, contrast values are ratios (e.g.*, *a contrast of 0.837 means the first group’s expected height is 83.7% of the second group’s height) because these values were back transformed from log transformations. Asterisks indicate the level of significance of the p-value as indicated below:*

********: P < 0.05 (significant at the 0.05 level)*

*********: P < 0.01 (significant at the 0.01 level)*

**********: P < 0.001 (highly significant at the 0.001 level)*

**Table S2.** Linear mixed effects models results for univariate models with and without severity included

*Note. m is the number of subsamples taken for each variable.* $\hat{\sigma}_{\left( {Block}_{i} \right)}$ *is the random block effect, and* $\hat{\sigma}_{\omega_{ij(k)}}$ *is the subsampling error. G1, G2, and G3 are the individual block effects for those respective three blocks in this study.* $x^{2}$ *is the chi squared value, and P is the p-value. Both marginal (Marg.) and conditional (Cond.) R^2^ values are displayed, along with Akaike Information Criterion (AIC) values. Asterisks indicate the level of significance of the p-value as indicated below:*

********: P < 0.05 (significant at the 0.05 level)*

*********: P < 0.01 (significant at the 0.01 level)*

**********: P < 0.001 (highly significant at the 0.001 level)*
