# Supplementary material for: Stable Isotopes Reveal the Drivers of Post‐Wildfire Natural Regeneration of Interior Douglas‐Fir Seedlings in British Columbia
Source: Ecol Evol. 2025 Mar 13;15(3):e71078. doi: 10.1002/ece3.71078 (PMC11906369; doi:10.1002/ece3.71078)
Supplement: Supplementary file 3 — Appendix S1. [file ECE3-15-e71078-s001.docx]

**Appendix A. Assessment of Burn Severity**

The Forest Analysis and Reporting Branch of the BC Ministry of Forests annually produces a vector fire severity map for British Columbia, employing the Burned Area Reflectance Classification (BARC) methodology developed by the USDA (Key and Benson 2006). This process involved gathering imagery from Landsat 8 or Sentinel-2 (depending on availability) before and after the 2017 fire season. The imagery was collected from June 1 to September 30, covering up to three years before the fire’s start date, and again from June 1 until the day of ignition in the fire year. Images as close to the fire's ignition date as possible were prioritized to ensure accuracy in reflecting initial ground conditions. Imagery collected for after the fire spans June 1 to September 30, either one or two years following the fire.

This imagery was used to produce a differenced Normalized Burn Ratio (dNBR) raster image, following this formula:

$$dNBR= \frac{({NIR}_{pre}-{SWIR}_{pre})}{{NIR}_{pre}+{SWIR}_{pre}}-\frac{({NIR}_{post}-{SWIR}_{post})}{{NIR}_{post}+{SWIR}_{post}}$$

Where ${NIR}_{pre}$ is the near-infrared (NIR) bands before the fire start date, ${SWIR}_{pre}$ is the short-wave infrared (SWIR) bands before the start date, ${NIR}_{post}$ is the NIR bands after the fire, and ${SWIR}_{post}$ is the SWIR bands after the fire.

The resulting dNBR raster is then classified according to the USFS BARC4 categories, using threshold values of 76, 110, and 187. Then, a vector dataset was created, followed by Euclidean distance smoothing.

Reference:

Key, C. H., & Benson, N. C. (2006). Landscape assessment (LA). In: Lutes, Duncan C.; Keane, Robert E.; Caratti, John F.; Key, Carl H.; Benson, Nathan C.; Sutherland, Steve; Gangi, Larry J. 2006. FIREMON: Fire effects monitoring and inventory system. Gen. Tech. Rep. RMRS-GTR-164-CD. Fort Collins, CO: US Department of Agriculture, Forest Service, Rocky Mountain Research Station. p. LA-1-55, 164.

**Appendix B.** Seedling biomass prediction

All statistical analyses were conducted using R version 4.3.2, and an alpha value = 0.05 was used as the significance level for all tests (R Core Team, 2023). A linear regression model was constructed using height and diameter measurements from the 81 subsamples with known biomass. The model, fitted using the 'lm()' function in R, includes interaction terms between height, diameter, and burn severity to account for variations in biomass prediction across different levels of burn severity. This model was:

$\hat{Y}_{i}=b_{0}+b_{1}*X_{1i}+b_{2}*X_{2i}+b_{3}*S_{{Mod}_{i}}+b_{4}*S_{{High}_{i}}+{b_{5}*X}_{1i}*S_{{Mod}_{i}}+{b_{6}*X}_{1i}*S_{{High}_{i}}+{b_{7}*X}_{2i}*S_{{Mod}_{i}}+{b_{8}*X}_{2i}*S_{{High}_{i}}$ (Eqn S1)

where $\hat{Y}_{i}$ is the predicted biomass (g), $b_{0}$ is the intercept, and $b_{1}$ and $b_{2}$ are the regression coefficients for the predictor variables $X_{1i}$ (height in cm) and $X_{2i}$ (diameter in mm), respectively. $b_{3}$ is the regression coefficient for the effect of moderate burn severity ($S_{{Mod}_{i}}$), $b_{4}$ is the regression coefficient for the effect of high burn severity ($S_{{High}_{i}}$), $b_{5}$ is the regression coefficient for the interaction between height and burn severity, and $b_{5}$ is the regression coefficient for the interaction between height and moderate burn severity, $b_{6}$ is the regression coefficient for the interaction between height and high burn severity, $b_{7}$ is the regression coefficient for the interaction between diameter and moderate burn severity, $b_{8}$ is the regression coefficient for the interaction between diameter and high burn severity. This model was applied to the 189 sampled seedlings lacking direct biomass measurements to predict biomass for these samples, and all further analyses on biomass were conducted using 270 observations (i.e., 189 predicted and 81 measured biomass values). The seedling biomass model had an R^2^ value of 0.759 and a root mean squared error (RMSE) of 1.085.

**Appendix C.** Linear Mixed Effects Models Without Burn Severity

We first built a linear mixed-effects modelwith the ‘lme’ function in the ‘nlme’ package in R (Pinhiero et al., 2023) with only fire severity as a fixed effect that follows the form below:

$\ln\left( {Biomass}_{ij(k)} \right)=\mu+R\left( {Block}_{i} \right)+T_{{Severity}_{j}}+\varepsilon_{ij}+\omega_{ij(k)}$ (Eqn S2)

Where ${Biomass}_{ij(k)}$ is biomass in grams for plot $k$ measured in block $i$ and severity $j$, $\mu$ is the overall mean of the logarithm of the response variable, $R\left( {Block}_{i} \right)$ is the random effect factor for block $i$, $T_{{Severity}_{j}}$is the fixed effect factor for burn severity $j$, $\varepsilon_{ijk}$ is the experimental error term, and $\omega_{ij(k)}$ is the subsampling error. ${Biomass}_{ij(k)}$ was log-transformed to meet the linear model assumption of normality, and post-hoc pairwise comparisons were performed with estimated marginal means using the Tukey method in the 'emmeans' function from the 'emmeans' package (Lenth 2023) for both Equation S2 and Equation S3.

This allowed us to determine that burn severity did have a significant effect on seedling biomass (*P* < 0.0001). We then used linear mixed effects models following the form of Equation 2 to determine what variables influenced biomass significantly while burn severity was also included as a fixed effect. However, some variables were related to burn severity (Table S1), so we also tested which variables were most important in affecting seedling biomass growth independent of burn severity. To accomplish this, we again used linear mixed effect models that followed this form:

$\ln\left( {Biomass}_{ij(k)} \right)= \beta_{0}+\beta_{1}X_{ij}+R\left( {Block}_{i} \right)+\varepsilon_{ij}+\omega_{ij(k)}$ (Eqn S3)

Where ${Biomass}_{ij(k)}$ is biomass in grams for block $i$, severity $j$ and subsample (plot) $k$ within each block $i$, $\beta_{0}$ is the intercept, $X_{ij}$ is each tested variable with slope parameter $\beta_{1}$, $R\left( {Block}_{i} \right)$ is the random effect factor for block $i$, $\varepsilon_{ij}$ is the experimental error term, and $\omega_{ij(k)}$ is the subsampling error.

The results of the models both including burn severity as a fixed effect (Equation 2) and excluding burn severity as a fixed effect (Equation S3) can be seen in Table S2. Canopy cover was the only variable that had a significant effect on biomass when burn severity was not included (*P* < 0.0001) but was not significant when burn severity was included (*P* = 0.4247). Leaf δ^13^C and stem water content had significant impacts on biomass whether burn severity was included or not (*P* < 0.013). All other variables were not significant in either model (*P* > 0.1).
